# Metal Load of Potentially Toxic Elements in Tuna (*Thunnus albacares*)—Food Safety Aspects

**DOI:** 10.3390/foods12163038

**Published:** 2023-08-12

**Authors:** József Lehel, Zita Papp, András Bartha, Péter Palotás, Rita Szabó, Péter Budai, Miklós Süth

**Affiliations:** 1 Department of Food Hygiene, University of Veterinary Medicine Budapest, István u. 2, H-1400 Budapest, Hungary; pappzita96@gmail.com (Z.P.); suth.miklos@univet.hu (M.S.); 2 National Laboratory for Infectious Animal Diseases, Antimicrobial Resistance, Veterinary Public Health and Food Chain Safety, University of Veterinary Medicine Budapest, István u. 2, H-1400 Budapest, Hungary; 3 Department of Animal Hygiene, Herd Health and Mobile Clinic, University of Veterinary Medicine Budapest, István u. 2, H-1400 Budapest, Hungary; bartha.andras@univet.hu; 4 The Fishmarket Fish Trading Company, Törökbálinti u. 23, H-2040 Budaörs, Hungary; palotas.peter@thefishmarket.hu; 5 Georgikon Campus, Institute of Plant Protection, Hungarian University of Agriculture and Life Sciences, Deák F. u. 16, H-8360 Keszthely, Hungary; szabo.rita@uni-mate.hu

**Keywords:** marine fish, heavy metal, tolerable intake of metal, consumer safety, environmental contaminants, potential exposure

## Abstract

The consumption of marine fishes has a positive effect on a consumer’s health; however, it poses a potential risk due to their level of heavy metals in their body. Heavy metals can be naturally found in the environment, but their concentration can be increased with anthropogenic activities. Samples of tuna (*Thunnus albacares*) were collected at a fishery market. The potentially toxic elements (arsenic, cadmium, lead, and mercury) were determined with a validated method in the flesh of fish using inductively plasma optical emission spectrometry after microwave digestion. Generally, the average concentration of them was below the official limit values regulated by the European Union, except for lead. Based on the concentrations of arsenic (inorganic derivates: 0.05 ± 0.02 mg/kg) and cadmium (0.03 ± 0.01 mg/kg) in the tuna fish samples, and their calculated EDI values (As: 0.03–0.09 µg/kg/day; Cd: 0.05–0.07 µg/kg/day), the investigated food could be declared safe for human consumption. Generally, mercury content was below the official regulated limit, and the calculated EDI value was below the dietary reference value (0.3 μg/kg/day) in most of the samples (90%), exceeding it only in two samples (0.69 and 0.82 μg/kg/day); thus, they may not be harmful to the consumer. The concentration of lead above the official maximum limit (0.30 mg/kg) in 40% of tuna samples (0.30–1.59 mg/kg), as well as the exceeding of the dietary reference value for lead (adult: 0.16 μg/kg/day; children: 0.26 μg/kg/day) based on the calculated EDI values (0.28–1.49 μg/kg/day), draw attention to the importance of environmental pollution and the protection of consumers’ health.

## 1. Introduction

Global fish production was about 179 million tons in 2018 including 82 million tons from aquaculture farms. Out of them, 156 million tons were marketed for human consumption, equivalent to annual, individual consumption as 20.5 kg/consumer, and the remaining amount was used for non-food purposes, e.g., to produce fishmeal and fish oil [1].

Basically, the food consumed by humans should contain essential components and nutrients (e.g., proteins, carbohydrates, lipids, vitamins, and macro- and microelements) to protect, to maintain, and to improve consumers’ health. However, they should be free of hazardous agents (environmental contaminants: arsenic, cadmium, lead, and mercury; industrial contaminants: dioxins and polychlorinated biphenyls; other chemicals: drugs and pesticides; physical contamination; etc.). Thus, the basic requirement of health protection is that the food should be safe, harmless, and fit for human consumption, and thus will be acceptable. Similarly, the food quality is also an important factor regarding acceptability, including nutritional–physiological values: composition and content, and sensory values: freshness, color, taste, smell, and texture [2].

Generally, the consumption of fishery products including aquatic organisms has outstanding significance in the nutrition of a population, especially in countries near a sea, using “local” natural-water, mainly marine fish, and their frozen and preserved fishery products, or farmed, aquaculture animals and their products, or their imported batches.

Fish, especially cold-water marine fish, are rich in n-3-polyunsaturated fatty acids (e.g., linolenic acid, eicosapentaenoic acid, and docosahexaenoic acid) that can reduce the potential risk of developing different diseases, e.g., diabetes, systemic arterial hypertension, obesity, hyperlipidemia, etc. [3,4,5].

Their high vitamin D content is essential to the absorption of calcium and to bone formation [6,7].

Similarly, they contain proteins including a rate of 15–20% with an almost full biological value (exceeds 90) and all essential amino acids (e.g., cysteine, methionine, etc.) necessary for different vital functions. Due to these components, the flesh of fish has high digestibility (85–95%) and it has beneficial effects on different health problems, e.g., metabolic syndrome, insulin sensitivity, skeletal muscle injury, etc. [3,8,9,10,11,12,13].

Due to their nutritional characteristics, fish can take up several chemicals (e.g., heavy metals) from their environment—dissolved, suspended, or emulsified in the milieu [14,15,16,17]. These chemicals can enter the fish body via the gill, gastrointestinal tract, or even through the skin [18,19,20,21]. They can be deposited or accumulated in different tissues, such as the muscle, liver, kidney, and gill, exceeding the regulated level [22,23,24,25,26,27,28]. Thus, fish can contain various chemical substances that can pose potential risks to consumers.

Based on COMMISSION REGULATION (EU) 2023/915, the maximum level of lead, cadmium, and mercury is 0.30 mg/kg, 0.10 mg/kg, and 1.0 mg/kg, respectively. The lead level refers to the muscle meat of fish and cadmium and mercury levels refer to tuna species (*Thunnus* spp., *Katsuwonus pelamis*, and *Euthynnus* spp.) [29].

The recommended tolerable intake levels for different metals have been determined as the Provisional Tolerable Daily, Weekly, or Monthly Intake (PTDI, PTWI, and PTMI) by the Joint FAO/WHO Expert Committee on Food Additives (JECFA) based on their maximum amount that cannot induce damage to health during long-life uptake to protect a consumer’s health. However, some of them have been withdrawn; thus, instead of it, the estimated daily intake (EDI) was calculated for each metal tested in our investigation [30,31,32,33,34].

The tolerance of environmental contaminants, thus that of metals, can be influenced with different factors, such as the concentration of the metal in the consumed food, the daily consumption of that food, and the body weight of the human. EDI is a parameter that was introduced to take these factors into account.

This study intended to measure the concentration of potentially toxic elements such as arsenic, cadmium, lead, and mercury in tuna species collected in a fishery market. Furthermore, the detected amounts were evaluated from the point of view of food safety, using the regulated limit of metals and their estimated daily intake level to assess the possibility of the potential risks of the consumption of these foodstuffs on consumers’ health. Generally, the flesh of fish was investigated and analyzed on the site of the harvesting by the researchers. The goal of this investigation was to assess the potential pollution load of potentially harmful elements to consumers far from a geographical origin.

## 2. Materials and Methods

### 2.1. Sampling

Yellowfin tuna as a total of 40 fish (*Thunnus albacares*) were collected from a fishery market in Hungary, and the fish originated from Sri Lanka, i.e., a FAO Fishing Area such as “Indian Ocean, Eastern” (Major Fishing Area 57), subarea 57.1 (Bay of Bengal).

The description of the area: “The waters bounded by a line commencing on the southeast coast of India at 77°00′ E longitude where the boundary between the States of Kerala and Tamil Nadu meet at the sea; thence due south to the Equator; thence due east to 80°00′ E longitude; thence due north to 3°00′ N latitude; thence due east to 85°00′ E longitude; thence due north to 5°00′ N latitude; thence due east to meet the northern coast of Sumatra; thence round the coast of Sumatra running south in the Strait of Malacca; thence across the Strait at 2°30′ N latitude to meet the coast of Malay Peninsula; thence in a northerly and westerly direction along the coasts facing the Bay of Bengal to the point of departure” [35].

Five grams of the flesh of fish was taken from the muscle of the back side of each animal using a metal-free tool. Then, they were placed into plastic bags properly labelled after shredding and homogenization (Potter S, B. Braun Biotech International GmbH, Melsungen, Germany). The samples were placed in a freezer until the analysis at a temperature of −70 °C (So-Low Ultra-Low Freezer, Model C85-9, Environmental Equipment Co. Inc., Cincinnati, OH, USA).

### 2.2. Analytical Method

The concentration of heavy metals in the fish samples was measured in the laboratory of the Department of Animal Hygiene, University of Veterinary Medicine, with inductively coupled plasma optical emission spectrometry (ICP-OES) [36].

#### 2.2.1. Reagents and Analytical Standards

Trace-analysis-quality nitric acid (69 m/m%, Aristar) and hydrogen peroxide (30 m/m%, Normapur) were applied for sample preparation; both were purchased from VWR International Ltd., Leicestershire, UK. Calibration was carried out with ICP multi- and mono-element standards acquired from Perkin Elmer Inc., Shelton, CT, USA and VWR International Ltd., Leicestershire, UK for quantitative ICP measurement. For determinations, argon gas was used with a 4.6 purity (Messer Hungarogáz Ltd., Budapest, Hungary). Quality controls (QC) were prepared using mussel tissue (ERM-CE278k) and tuna fish (ERM-CE464) originated from the European Commission, Joint Research Centre, Geel, Belgium.

#### 2.2.2. Preparation of Sample

The sample (0.5 g) was weighed on a CEM MARS XPreSS teflon vessel and the decomposition was induced in a CEM MARS6 microwave digestion system (CEM Corporation, Matthews, NC, USA) after adding 5 mL of both nitric acid and of hydrogen peroxide. The process was performed with the following parameters: ramp: 35 min; temperature: 200 °C; hold: 50 min; and energy: 1700 W. After the digestion, deionized water was added to the sample, up to 25 mL. Then, the sample was diluted with deionized water twice using a 1 mg/L Y solution as an internal standard and a 0.25 mg/L Au solution (VWR International Ltd., Leicestershire, UK) for the stabilization of mercury content. Finally, the prepared samples were analyzed with ICP-OES. The preparation of the blank and the quality control (QC) samples was performed with the same process.

#### 2.2.3. Instruments

The determination of the potentially toxic elements was performed with an ICP-OES instrument (Perkin Elmer Optima 8300 DV, Perkin Elmer, Shelton, CT, USA) including the following parameters: RF generator: 40 MHz solid state, free running, flat plate plasma technology; RF power: 1300 W; Nebuliser type: BURGENER PEEK MIRA MIST; plasma gas flow rate: 12 dm^3^/min; auxiliary gas flow rate: 0.2 dm^3^/min; Nebuliser gas flow rate: 0.7 dm^3^/min; and observation height: 15 mm.

#### 2.2.4. Validation Process

Based on the force guideline, different validation parameters were established to assess the reliability of the analytical method and sample preparation (Table 1) [37]. The limit of detection (LOD) and limit of quantitation (LOQ) were determined as 3 and 10 times the standard deviation (SD) of the signals of the blank samples, respectively. Precision was calculated as the relative SD of the signals from ten replicates of the same sample. Trueness was determined by analyzing standard tissue materials (mussel tissue ERM-CE278k and tuna fish ERM-CE464). After that, the solution of the four investigated elements with a known concentration (50 µg/kg each) was added to the certified mussel and tuna tissues and the results of these measurements were compared and evaluated, and expressed in percentages.

Precision was accepted below 20%; trueness was adequate if the deviation of the measured parameter did not exceed ±15%. Linearity was evaluated with the equations of the calibration curves. The matrix effect was not studied since the Y solution used as the internal standard provided compensation. The results of the QC samples and the recovery percentages are summarized in Table 2.

### 2.3. Exposure Calculation

Based on the EFSA data, the average annual fish consumption per person is 23.97 kg/year/person in the European Union; thus, the 65.7 g/day/person value was applied during the calculation [38].

For the calculation of the estimated daily intake (EDI) [34], the concentrations of heavy metals (arsenic, cadmium, lead, and mercury) in the investigated samples were multiplied with the daily average fish consumption (65.7 g/day/person) [38], and then these amounts were divided with an average human body weight (70 kg).

### 2.4. Statistical Evaluation

A statistical analysis was carried out with Microsoft Excel and the R program (version 3.3.2.).

During the statistical evaluation, half of the LOD value was used in the case of those samples in which the concentration of metals was below the limit of detection [39].

The calculated EDI values based on the detected concentrations of the investigated metals were compared to the dietary heavy metal reference values [30,31,32,33,40,41].

## 3. Results and Discussion

The concentrations of metals are presented in Table 3.

### 3.1. Arsenic

The arsenic concentration of all samples was above the LOD (100%). The mean concentration of As in this investigation was 0.98 ± 0.47 mg/kg of wet weight (w.w.). Generally, most arsenic (95%) is found in marine fish as organic forms (e.g., arsenobetain, arsenocholine, and arsenosugars); however, they are less toxic and are rapidly eliminated from a human organism, and thus they are of much lesser importance regarding food safety aspects [42,43,44]. Thus, the maximum limit of As in fish species is not regulated. However, the remaining amount (5%) is incorporated as inorganic derivatives that may have possible toxic effects. Based on the calculation, the concentrations of inorganic arsenicals were between 0.03 and 0.10 mg/kg (mean: 0.05 ± 0.02 mg/kg w.w.) in our study (Table 3).

The detected As level of 1.30 ± 0.34 mg/kg w.w. in the flesh of a tuna species (*Thunnus* spp.) in Aracaju, Sergipe, north-eastern Brazil [45], and that of two tuna species (*Thunnus obesus* and *Thunnus thynnus*) in their flesh collected from Japanese restaurants in the Republic of Korea [46], are similar to our findings (0.98 ± 0.47 mg/kg w.w.).

Núñez et al. (2018) [47] measured a higher As concentration (3.78 ± 2.24 mg/kg w.w.) in the flesh of yellowfin tuna in Galicia, North-West Spain. The total As concentration was similar, such as 3.47 ± 0.21 mg/kg d.w., in the flesh of yellowfin tuna at Jakarta Fishing Port, Indonesia [48].

The results were almost the same (2.24 ± 0.66 mg/kg dry wet (d.w.)) that were measured by Ruelas-Inzunza et al. (2018) [49] in the flesh of yellowfin tuna in the eastern area of the Pacific Ocean around Mexico. In these samples, the concentration was higher in the liver (72 ± 5.71 mg/kg d.w.).

However, As contamination was lower in the flesh of bluefin tuna (*Thunnus thynnus*), such as 0.03 mg/kg w.w. (interval: 0.02–0.59 mg/kg w.w.) in Spain at the Canary Islands [50].

Barreca et al. (2023) measured a lower As level (0.003–0.085 mg/kg) in different typical Mediterranean diets containing several fish species (salmon, dolphinfish, anchovies, tuna, and sardines) and other components (pasta and vegetables) compared with our results measured in the flesh of fish. They stated that their findings are in good agreement with the average daily consumption of As in European Member States, and that their results are below the tolerable maximum daily intake (2 µg/kg b.w.) set by EFSA using the consumption of a single portion (100 g) of a Mediterranean meal, and they have no potential risks to consumers [51].

Compared to the dietary reference value of arsenic (0.3 μg/kg/day), the calculated EDI for arsenic was below it (100%) in all investigated samples in the case of inorganic arsenicals (Table 4) [30,34,40].

### 3.2. Cadmium

The concentration of Cd was detected above the LOD in 20% of samples and all of them (100%) were below the maximum levels (0.1 mg/kg) regulated for tuna fish in the European Union (Table 3) [29].

In different fishing areas and in the fishery products, the detected concentrations of Cd are closely similar to our findings (0.03 ± 0.01 mg/kg w.w.).

The level of Cd was 0.01 ± 0.01 mg/kg w.w. and 0.02 ± 0.02 mg/kg w.w. in the flesh and the tissue of the belly area of yellowfin tuna in Sri Lanka at the Galle, Mutwal, Negombo, and Trincomalee areas and in the fish collected from a fish market in Sri Lanka [52,53]. Its level was slightly elevated (<0.01–0.13 mg/kg w.w.) in 2019 [54].

The amount of Cd was detected as 0.02 ± 0.01 mg/kg w.w. in the flesh of yellowfin tuna in Galicia, North-West Spain [47].

The mean concentration of Cd was 0.03 ± 0.03 mg/kg in the fishery products of different tuna fish (skipjack (*Katsuwonus pelamis*), yellowfin tuna, and bigeye tuna (*Thunnus obesus*)) in Ecuador [55].

The measured quantity of Cd was 0.08 ± 0.02 mg/kg w.w. in the flesh of tuna in Aracaju, Sergipe, north-eastern Brazil [45].

The detected Cd level in the flesh of yellowfin tuna sampled in Foggia, Italy was 0.01 ± 0.01 mg/kg w.w., and that of skipjack tuna was 0.03 ± 0.01 mg/kg w.w. [56].

Similarly, a smaller concentration (mean: 0.01 mg/kg w.w.; interval: 0.00–0.13 mg/kg w.w.) was detected in the flesh of another tuna species, bluefin tuna in Spain at the Canary Islands [50].

However, the mean concentration of Cd was 0.25 ± 0.21 and 0.23 ± 0.20 mg/kg w.w. in the flesh of yellowfin tuna at the western coast of the Indian Ocean, Mozambique Channel and in waters surrounding Reunion Island. The amount of Cd was greatly increased in the liver of the fish (138 ± 60 and 126 ± 130 mg/kg w.w.) compared to the muscle. Its amount was a bit higher in skipjack (muscle: 0.61 ± 0.37 mg/kg w.w.; liver: 153 ± 95 mg/kg w.w.) at Reunion Island [57].

The Cd level in the flesh of yellowfin tuna sampled from the Eastern Pacific Ocean, the city of Manta, Ecuador was 2.40 ± 5.10 mg/kg w.w. Its quantity was higher (13.00 ± 10.00 mg/kg w.w.) in the liver of the fish [5].

The Cd concentration was between 0.0006 and 0.005 mg/kg in Mediterranean meals containing several fish species (salmon, dolphinfish, anchovies, tuna, and sardines), pasta, vegetables, and other components. Based on the average consumption (100 g of a Mediterranean diet), these meals did not pose a potential hazard exceeding the tolerable weekly intake [51]. Our results were higher (0.03 ± 0.01 mg/kg), but it was investigated in the flesh of tuna only.

The calculated EDI values of Cd using the investigated samples were between 0.05 and 0.07 μg/kg/day and all of them were below the dietary reference value of them (1 μg/kg/day) (Table 4) [31,34,40].

### 3.3. Mercury

The mercury concentration in most of the samples (90%) was below the LOD (0.5 mg/kg); thus, a statistical analysis was not performed. The detected amount of it was below the official regulated limit of mercury (1.0 mg/kg) in all investigated samples of tuna species (Table 3).

Similar to our findings, different researchers detected mercury below the official regulated limit.

The Hg level of the flesh of yellowfin tuna was 0.30 ± 0.14 mg/kg w.w. in Sri Lanka at the Galle, Mutwal, Negombo, and Trincomalee areas in 2013 [52]. Basically, it did not increase until 2019 at these areas (mean: 0.30 mg/kg w.w.) [58]. Likewise, a low level of mercury (0.39 ± 0.19 mg/kg w.w.) was measured in the belly area of yellowfin tuna collected from a fish market in Sri Lanka [53]. However, in some samples of yellowfin tuna, Hg was above the official limit in the Indian Ocean at Sri Lanka [54].

Ruelas-Inzunza et al. (2018) [49] measured a 0.71 ± 0.62 mg/1 d.w. Hg concentration in the flesh of yellowfin tuna in the eastern area of the Pacific Ocean around Mexico. Its concentration was higher in the liver samples (0.88 ± 0.77 mg/kg d.w.).

The mercury level of the flesh of yellowfin tuna captured at Jakarta Fishing Port, Indonesia was 0.68 ± 0.08 mg/kg d.w. [48].

The detected level of Hg was 0.60 ± 0.15 mg/kg w.w. in the flesh of tuna in Aracaju, Sergipe, north-eastern Brazil [45].

The measured Hg concentration in the flesh of yellowfin tuna collected in Foggia, Italy was 0.55 ± 0.43 mg/kg and that of skipjack tuna was 0.40 ± 0.31 mg/kg [56].

The quantity of Hg was below the official limit—0.56 ± 0.38 mg/kg w.w. and 0.65 ± 0.52 mg/kg w.w.—in the flesh of yellowfin tuna in the Western Indian Ocean, Mozambique Channel and in waters surrounding Reunion Island, and it was 0.67 ± 0.26 mg/kg w.w. in skipjack at Reunion Island. However, its concentration in the liver of these species was 1.15 ± 2.30 mg/kg w.w. and 3.27 ± 8.11 mg/kg w.w., and it was unobjectionable in the liver of skipjack (0.51 ± 0.28 mg/kg w.w.) [57].

The interval of the Hg level in the flesh of yellowfin tuna caught from the Pacific Ocean (FAO Fishing Zone 71) was 0.10–0.35 mg/kg w.w. and it was 0.01–0.23 mg/kg in the Indian Ocean (FAO Fishing Zone 57) [3].

The average level of Hg was 0.23 ± 0.14 mg/kg w.w. in the fishery products of different tuna species (skipjack, yellowfin tuna, and bigeye tuna) in Ecuador [55].

The mean Hg concentration in the flesh of bluefin tuna was 0.47 mg/kg w.w. in Spain at the Canary Islands [50].

The measured quantity of Hg was 0.47 mg/kg w.w in the flesh of Atlantic bluefin tuna collected from commercial centers in the Apulia region, Italy [59].

The detected average Hg concentration in the flesh of yellowfin tuna sampled from the Eastern Pacific Ocean, the city of Manta, Ecuador was above the official regulated limit, such as 1.40 ± 1.30 mg/kg w.w. Its quantity was highly elevated in the liver sample of fish, such as 2.0 ± 2.6 mg/kg w.w. [60].

However, elevated Hg levels were measured in the flesh of different tuna species over the regulated limit in the Ionian Sea (0.49–1.60 mg/kg w.w.) and Atlantic Ocean (0.008–1.3 mg/kg w.w.) [61,62].

The concentration of Hg was between 0.0001 and 0.035 mg/kg in Mediterranean meals (pasta with different fish species and vegetables), and the authors stated that the consumption of their single portion (100 g) is in good agreement with EFSA findings, and the tolerable weekly intake is not exceeded; thus, they have no potential risks to human consumers [51].

The calculated EDI value of Hg using the half of the LOD value (similarly to the statistical evaluation) was below the dietary reference value (0.3 μg/kg/day) in most of the samples (90%) (0.23 μg/kg/day). The EDI value was higher only in two samples (0.69 and 0.82 μg/kg/day); however, the detected concentration of Hg was below the maximum level (1.0 mg/kg) in all investigated samples; thus, basically, they did not pose a potential harmful effect to a consumer [32,34,41].

### 3.4. Lead

Among the investigated tuna samples, 47.5% of the measured quantity was below the LOD (0.2 mg/kg), and the detected amounts were between 0.3 and 1.59 mg/kg; however, 40% of them were above the regulated limit value in tuna species (0.3 mg/kg) (Table 3).

Based on the scientific literature, the measured values of Pb were below the official permitted concentration limit.

The lead level in the flesh of yellowfin tuna was 0.09 ± 0.12 mg/kg w.w. in Sri Lanka at the Galle, Mutwal, Negombo, and Trincomalee areas [52]. Similar concentrations were detected in the tissue of the belly area of yellowfin tuna collected from a fish market in Sri Lanka (0.06 ± 0.06 mg/kg) [53].

The quantity of Pb was detected as 0.09 ± 0.06 mg/kg w.w. in the flesh of tuna in Aracaju, Sergipe, north-eastern Brazil [45].

The measured Pb level in the flesh of yellowfin tuna collected from the Eastern Pacific Ocean, the city of Manta, Ecuador was 0.07 ± 0.06 mg/kg w.w. Its level was similar in the liver sample of the fish, such as 0.09 ± 0.07 mg/kg w.w. [60].

The average Pb concentration was 0.06 ± 0.05 mg/kg w.w. in the fishery products of different tuna species (skipjack, yellowfin tuna, and bigeye tuna) in Ecuador [55].

The measured Pb level in the flesh of yellowfin tuna collected at the western coast of the Indian Ocean, Mozambique Channel and in waters surrounding Reunion Island was 0.09 ± 0.14 and 0.02 ± 0.07 mg/kg w.w. Its level was almost the same in the liver sample of the fish, such as 0.13 ± 0.12 and 0.05 ± 0.08 mg/kg w.w. A similar amount of it was detected in skipjack (muscle: 0.07 ± 0.08 mg/kg w.w.; liver: 0.12 ± 0.14 mg/kg w.w.) at Reunion Island [57].

The measured Pb concentration in the flesh of yellowfin tuna sampled in Foggia, Italy was 0.02 ± 0.03 mg/kg w.w., and that of skipjack was 0.01 mg/kg w.w. [56].

The average Pb level in the flesh of bluefin tuna was 0.004 mg/kg w.w. in Spain at the Canary Islands [47], and that of the same species was 0.11 mg/kg w.w. in the Apulia region, Italy [59].

Pb concentrations in the investigated Mediterranean meals containing pasta, vegetables, and several fish species (salmon, dolphinfish, anchovies, tuna, and sardines) were between 0.005 and 0.053 mg/kg, which are lower than our results measured only in the flesh of tuna. The authors described that the consumption of a single portion (100 g) of these meals resulted in no potential risks to consumers [51].

The calculated EDI values of Pb using the investigated samples were between 0.19 and 1.47 μg/kg/day and all of them (100%) were above the dietary reference value for adults (0.16 μg/kg/day) and for children (0.26 μg/kg/day) (Table 4) [33,34,40].

## 4. Conclusions

Due to international trade and transport, caught fish and different fishing products can reach different countries, and the pollution and contaminants in them can cause health problems not only in coastal countries (where the fishing took place) but they also can occur worldwide, in any country to which the given product reaches. Based on the per capita fish consumption data of European countries, it can be stated that the amount of annual fish consumption in those countries that do not have maritime areas (e.g., in Hungary, it is 6.28 kg) is below the European average (23.97 kg). However, the fish consumption may even be over 2–3 times higher than the European average in the maritime countries (e.g., in Portugal, it is 59.91 kg) [38]. Considering the significant differences in per capita fish consumption data, it is justified to use an average daily dietary intake value during risk assessment in order to protect the health of those who regularly consume larger quantities of the given food. In our study, the possible metal load (arsenic, cadmium, lead, and mercury) based on an average consumption was examined and calculated.

The concentrations of As is not objectionable because the majority of it is found as organic derivatives, and the calculated amount of inorganic derivatives was only 0.03–0.10 mg/kg, and its calculated EDI value (0.03–0.09 µg/kg/day) does not pose a harmful effect to consumers compared to the previous recommended value (0.3 µg/kg/day).

The Cd contents of the samples (0.03 ± 0.01 mg/kg) were not objectionable because the detected concentration was below the regulated limit (0.10 mg/kg) in all samples, and the calculated EDI values (0.05–0.07 µg/kg/day) were below the reference value (1 µg/kg/day).

The measured levels of mercury (0.50–0.88 mg/kg) were below the official regulated limit (1.0 mg/kg) in all investigated tuna samples (100%), and the calculated EDI value was below the dietary reference value (0.3 μg/kg/day) in most of the samples (90%), exceeding it only in two samples (0.69 and 0.82 μg/kg/day). Basically, they could not induce a harmful effect in human consumers.

The lead concentrations were above the regulated limit (0.30 mg/kg) in 40% of tuna samples (0.30–1.59 mg/kg); thus, these may be harmful to consumers exceeding the reference value for adults (0.16 μg/kg/day) and for children (0.26 μg/kg/day) compared with calculated EDI values (0.28–1.49 μg/kg/day).

Based on the concentrations of arsenic, cadmium, and mercury in the tuna fish samples, and their calculated EDI values, the investigated food could be declared safe for human consumption. However, the concentration of lead above the official maximum limit, as well as the exceeding of the dietary reference value for lead, draw attention to the importance of environmental pollution and the protection of consumers’ health through the provision of the “Farm to fork” and “One Health” concepts. The long-term intake of foods that are relatively lightly contaminated with heavy metals can contribute to the development of chronic diseases due to the accumulation property of metals.

## Figures and Tables

**Table 1:** Results of validation.

Element	Wavelength of Detection (nm)	Calibration Curve Parameters			Limit of Quantitation (mg/kg)	Limit of Detection (mg/kg)	Precision (%)	Trueness (%)
		Equation (y = a·x + b) (1)		(2)				
		a	b	r				
Arsenic	188.979	1287	0	0.999828	1.67	0.50	12.7	13.6
Cadmium	228.802	63,870	0	0.999529	0.17	0.05	8.4	−10.9
Mercury	194.168	10,030	0	1.000000	1.67	0.50	12.3	8.1
Lead	220.353	6520	0	0.999813	0.67	0.20	3.5	−8.4

(1) ‘y’ means the signal of the target element at the given concentration level; ‘x’ means the concentration; (2) regression coefficient.

**Table 2:** Results of quality control (QC) measurement (mg/kg).

Element	Certified Value	Measured Value	LOD	Recovery (%)
ERM-CE287k (mussel tissue)				
Arsenic	6.70	6.87 ± 0.08	0.50	102.5
Cadmium	0.34	0.36 ± 0.02	0.05	106.5
Lead	2.18	2.00 ± 0.11	0.20	91.7
ERM-CE464 (tuna fish)				
Mercury	5.24	5.14 ± 0.08	0.50	98.1

**Table 3:** Quantity of potentially toxic elements (mg/kg w.w.) in the flesh of yellowfin tuna (*Thunnus albacares*).

Concentration	As		Cd	Hg	Pb
	Total	Inorganic (5% of Total)			
Mean ± SD	0.98 ± 0.47	0.05 ± 0.02	0.03 ± 0.01	NA	0.39 ± 0.37
Minimum (LOD)	0.50	0.03	0.05	0.50	0.20
Minimum (measured)	0.51	0.03	0.05	0.73	0.30
Maximum (measured)	2.05	0.10	0.07	0.88	1.59
Regulated limit	-	-	0.10	1.00	0.30

NA = Not Applicable, because 95% of the detected concentration is <LOD.

**Table 4:** Dietary heavy metal reference values (μg/kg/day) and the calculated estimated daily intake of metals (μg/kg/day).

	As (Inorganic, 5% of Total As)	Cd	Pb
Reference value	0.3	1	0.16 (adults) 0.26 (children)
**Estimated daily intake**			
Average	0.05	0.03	0.37
Minimum (LOD)	0.03	0.05	0.19
Minimum (measured)	0.03	0.05	0.28
Maximum (measured)	0.09	0.07	1.49
Ratio of sample above the reference value (%)	0	0	100

## Data Availability

The data used to support the findings of this study can be made available by the corresponding author upon request.
